# A New Unsupervised Machine Learning Method for Metabarcoding Analysis Based on Selected Mock Community Data

**DOI:** 10.34133/csbj.0221

**Published:** 2026-09-22

**Authors:** Hyung-Eun An, Min-Ho Mun, Jung-Il Kim, Chang-Bae Kim

**Affiliations:** ^1^ Department of Biotechnology, Sangmyung University, Seoul 03016, Republic of Korea.; ^2^ Institute of Intelligence Informatics Technology, Sangmyung University, Seoul 03016, Republic of Korea.; ^3^ Division of Biodiversity Conservation, Honam National Institute of Biological Resources, Mokpo 58762, Republic of Korea.; ^4^ Ocean Climate Response and Ecosystem Research Department, Korea Institute of Ocean Science and Technology, Busan 49111, Republic of Korea.

## Abstract

DNA metabarcoding analysis has emerged as a powerful tool for biological monitoring, enabling the identification and assessment of biodiversity across ecosystems. Conventional analytical methods, including operational taxonomic unit (OTU) clustering and amplicon sequence variant (ASV) analysis, have demonstrated strengths and limitations, including bias in estimating biodiversity and challenges in detecting rare sequences. To address these challenges, we developed unsupervised machine learning (UML)-based methods utilizing hierarchical density-based spatial clustering of applications with noise (HDBSCAN) and ordering points to identify the clustering structure. This study focused on 3 genetic markers: 12S rRNA, cytochrome c oxidase subunit I, and 18S rRNA. Mock community datasets comprising diverse taxa were analyzed using UML-based methods, OTU clustering, and ASV analysis. Nineteen biological feature descriptors were evaluated across all datasets. Nucleic acid composition and composition of k-spaced nucleic acid pairs consistently produced the best clustering performance. A comparative analysis of the clustering methods was performed. HDBSCAN produced relative abundance profiles broadly consistent with OTU clustering and ASV analysis. Additionally, comparisons of sensitivity and precision based on detected species demonstrated that UML-based methods, especially HDBSCAN, provided a favorable balance between false-positive and false-negative detections in several datasets, suggesting their promise as alternative clustering approaches for DNA metabarcoding. However, their applicability to real environmental systems remains to be validated.

## Highlights

• A novel unsupervised machine learning (UML)-based metabarcoding analysis method using HDBSCAN and OPTICS was developed
• The UML-based approach shows improved performance, with NAC (nucleic acid composition) and CKSNAP (composition of k-spaced nucleic acid pairs) feature representations producing well-separated clusters with superior clustering performance indices across multiple genetic markers
• HDBSCAN showed similar results to OTU and ASV methods in relative abundance estimation while reducing false-positive and false-negative species detections
• The proposed UML-based method provides a flexible alternative for improving biodiversity analysis using metabarcoding

## Graphical Abstract

**Figure:**
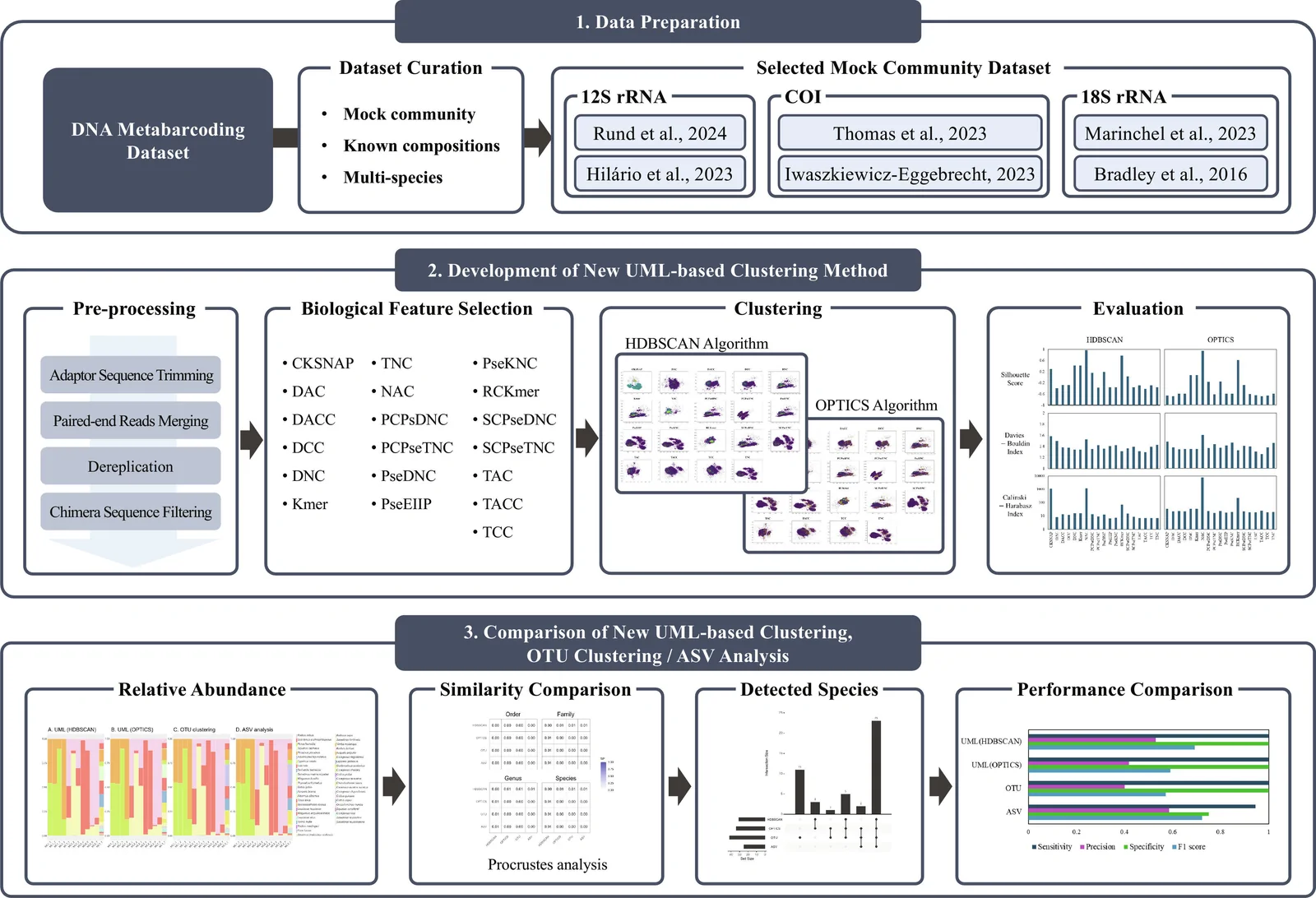

## Introduction

DNA metabarcoding, a method of biodiversity monitoring, is a molecular technique that can identify multiple organisms simultaneously using DNA extracted from a single environmental sample [1–3]. This technique adopts polymerase chain reaction (PCR) and high-throughput sequencing with universal primers to amplify a genetic barcode, defined as a short, standardized DNA region used for taxonomic identification, from the DNA in an environmental sample. To understand biodiversity using DNA metabarcoding, various taxonomic groups across diverse ecosystems, including fish and benthic invertebrates in aquatic environments, as well as organisms in terrestrial and airborne environments, are commonly analyzed. Earlier studies utilizing DNA metabarcoding demonstrated its effectiveness in monitoring environmental biodiversity [4,5]. In particular, environmental DNA (eDNA) metabarcoding has been widely recognized as a comprehensive and noninvasive approach for biodiversity assessment in aquatic systems that permits the detection of multiple taxa from water samples [2,6]. Beyond aquatic environments, this approach has been successfully applied to a broad range of environmental samples, including soil, air, and sediment, highlighting its versatility in surveying both terrestrial and aquatic communities [1]. Furthermore, DNA metabarcoding has been widely applied to diet analysis using fecal or gut-derived eDNA, enabling high-resolution detection of trophic interactions [7–9]. These applications have contributed to a better understanding of food webs and ecosystem functioning. In addition, its applications extend to terrestrial arthropod biomonitoring, diet analysis, airborne vertebrate surveys, soil-based biodiversity assessment, rare species conservation, invasive species surveillance, and ecological status assessment [10–15]. Because of these methodological advantages, DNA metabarcoding has substantially improved our ability to characterize biodiversity and uncover biological interactions across ecosystems. DNA metabarcoding generates large volumes of sequence data that provide valuable insights into biodiversity; however, advanced analytical approaches and bioinformatic tools are required to accurately interpret these complex datasets [16,17].

Analyses of DNA metabarcoding data began with the removal of adapter sequences, low-quality reads, and chimeric sequences from raw sequence data, followed by the merging of paired-end reads and progressing through the generation of unique sequence variants to taxonomic assignment [18]. In metabarcoding data analysis, various approaches can be used to group similar reads into representative sequence units. Among these, operational taxonomic unit (OTU) clustering and amplicon sequence variant (ASV) analysis are most commonly applied [19–21]. OTU clustering groups similar sequences into clusters, from which a representative sequence is derived [19]. The representative sequence of an OTU is typically selected as the cluster centroid, although consensus-based approaches can also be used depending on the implementation [22]. OTU clustering-based methods have traditionally been used commonly to analyze metabarcoding data [19]. OTU clustering relies on a predefined similarity threshold (most commonly 97%) to group sequences into clusters. However, this fixed threshold does not account for variation in intraspecific and interspecific sequence divergence across taxonomic groups and genetic markers, potentially causing a single species to be split across multiple OTUs or distinct species to be merged into one, leading to over- or underestimation of true diversity [20,23,24]. Genetically similar variants are grouped into clusters, limiting the capacity of OTUs to detect subtle differences in diversity and composition within samples [20,24]. Conversely, ASV analysis provides a more advanced approach by differentiating variants based on single-nucleotide changes, often permitting species-level identification, although this depends on the genetic marker and taxonomic group [19]. These results are accurate up to single-nucleotide differences, and they can produce sequences that are directly comparable across studies [25,26]. The ASV approach employs a statistical error model to distinguish true biological sequences from those generated by sequencing errors, enabling single-nucleotide resolution without a predefined similarity threshold. However, this error-modeling framework is platform- and run-specific, requiring separate model training for each sequencing run. Additionally, intraspecific sequence variation that escapes the error model might be retained as distinct ASVs, potentially overestimating true species diversity [27]. Both OTU- and ASV-based methods are widely used in current metabarcoding studies, and ASV-based methods have been increasingly adopted owing to their reproducibility and higher resolution [22].

Furthermore, although low-abundance sequences are often treated as potential artifacts in conventional metabarcoding analyses, accumulating evidence suggests that rare variants, including components of the rare biosphere or early signals of environmental change, might represent ecologically meaningful taxa [28,29]. However, approaches that rely on strict clustering thresholds or error-filtering frameworks might inadvertently remove or merge such variants, potentially eliminating biologically relevant information. Consequently, analytical approaches that can distinguish true biological signals from noise without excessively discarding rare sequences are needed. In addition, both OTU and ASV analyses require researchers to define multiple parameter settings, a challenging endeavor that might increase the barrier to entry for biodiversity research using DNA metabarcoding [30]. Accordingly, alternative methods that can improve both analytical accuracy and usability in metabarcoding studies are urgently needed [2,30–33].

To address this issue, machine learning involving algorithms that can learn and make decisions without explicit user guidance can be applied. Machine learning methods, including DNA metabarcoding [34–36], DNA polymorphism analysis [37], and species delimitation [38], have found recent applications in biology [39,40]. Machine learning combined with DNA technology can handle big data and complex tasks by learning from datasets and improving performance based on empirical analysis [41–43]. Recent benchmarking of machine learning approaches for environmental metabarcoding has further demonstrated that analytical performance can vary according to dataset characteristics and model selection [44]. In particular, unsupervised machine learning (UML) algorithms have proven valuable in revealing the underlying structure in datasets without prior information [38]. UML has displayed utility in research areas such as metabarcoding analysis, in which the clustering of data samples progresses using only inherent data structures without predefined hypotheses regarding models, species number, sample assignments, population parameters, or divergence levels [38,45,46]. Thus, UML can be appropriately applied to analyze an undetermined number of clusters in DNA metabarcoding data analysis. For instance, a UML-based method was used to analyze microbiome metabarcoding data to characterize and cluster subpopulations [47].

Various UML algorithms have been adopted for unsupervised clustering [38]. Among these, hierarchical density-based spatial clustering of applications with noise (HDBSCAN) is used to cluster data points based on their density distribution in high-dimensional space [48,49]. Unlike traditional clustering algorithms, HDBSCAN can discover clusters of varying shapes and sizes and identify noise points [49]. HDBSCAN operates by creating a hierarchy of clusters and determining the most stable and meaningful clusters in a set of data, making it particularly useful for complex datasets with irregularly shaped clusters and varying densities [50]. Similarly, ordering points to identify the clustering structure (OPTICS) is used to identify density-based clusters in spatial data [51]. Unlike traditional clustering algorithms, OPTICS can identify meaningful clusters in data with varying densities and shapes. The algorithm linearly orders the data points such that the spatially closest points are neighbors in the ordering, and it stores a special distance value for each point to represent the density required for clustering. This information is often visualized as a reachability plot, providing a hierarchical view of the clustering structure [52].

HDBSCAN has been applied to 16S rRNA-based microbial community analysis, enabling species-level bacterial identification and relative abundance profiling [53]. Similarly, OPTICS has been utilized for biological sequence clustering, demonstrating robust performance in grouping protein sequences based on sequence similarity [52]. These prior applications of density-based clustering algorithms in sequence-based biological analyses suggest their potential for metabarcoding, in which amplicon sequences must be grouped into biologically meaningful clusters for downstream taxonomic assignment.

**Fig. 1.**
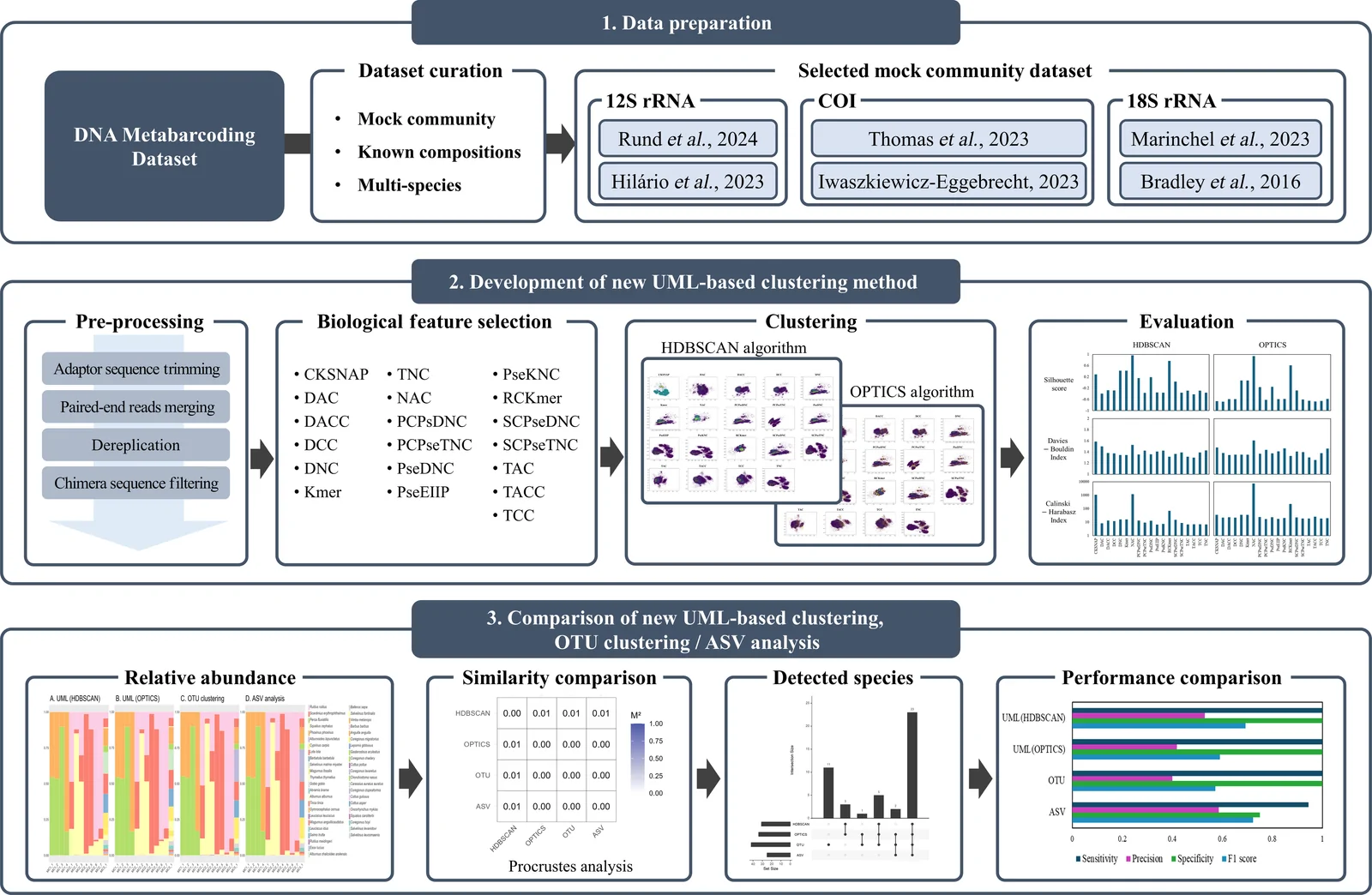
Schematic overview of this study. The analysis consisted of 3 main procedures: (1) data preparation; (2) development of a new unsupervised machine learning (UML)-based clustering method; and (3) comparison of the new UML-based methods with operational taxonomic unit (OTU) clustering and amplicon sequence variant (ASV) analysis.

In this study, we aimed to identify UML-based methods capable of analyzing complex datasets without predefined hypotheses to enhance the accuracy of biodiversity assessments across multiple ecological taxa. The study specifically investigated 3 key genetic markers: 12S rRNA for fish, cytochrome c oxidase subunit I (COI) for benthic invertebrates, and 18S rRNA for eukaryotic organisms. For this purpose, we utilized mock community datasets comprising diverse taxa to evaluate the performance of the UML-based methods compared with established approaches. Then, relative abundance and detected species were analyzed and compared among the newly developed UML-based methods, OTU clustering, and ASV analysis. These analyses were performed on identical datasets to ensure consistent comparisons across methods and comprehensively assess the advantages and limitations of each approach. A schematic diagram outlining the overall workflow of this study is provided in Fig. 1.

## Materials and Methods

### Dataset construction for developing new UML-based methods

To develop and evaluate novel UML-based methods for DNA metabarcoding analysis (Fig. 1), 6 publicly available mock community datasets were selected, comprising 2 datasets per genetic marker (12S rRNA, COI, and 18S rRNA). Mock communities are artificially constructed samples containing DNA from multiple known species, and their predetermined species identities provide a reliable reference framework for benchmarking analytical methods. Raw sequencing data (FASTQ format) for all 6 datasets were retrieved from the NCBI Sequence Read Archive (https://www.ncbi.nlm.nih.gov/sra) or the repositories provided in the original studies (Table S1). Details of the literature search strategy and computational infrastructure used in this study are provided in Supplementary Materials and Methods S1.

### Preprocessing metabarcoding sequences

The UML-based methods and OTU clustering shared an identical preprocessing pipeline, whereas the ASV analysis followed a distinct pipeline based on the DADA2 error-modeling framework (Supplementary Materials and Methods S2). For UML and OTU-based analyses, raw reads were processed sequentially via quality trimming (Q30), adapter and primer removal, paired-end read merging, low-quality sequence filtering, dereplication, and chimera filtering, using QIIME2 version 2024.2.0 [54], VSEARCH version 2.22.1 [55], and cutadapt version 4.6 [56]. For ASV analysis, raw reads were quality-trimmed (Q30) and processed using DADA2 [25] implemented in QIIME2, with truncation lengths determined per marker and study based on read quality profiles.

### Biological feature extraction for the UML-based methods

Preprocessed metabarcoding sequences were represented as fixed-length feature vectors using feature-encoding algorithms. Unlike conventional OTU and ASV approaches, which operate directly on sequence similarity or sequence-level variation, the proposed UML framework utilizes biological feature descriptors as the primary representation of sequence information. As density-based UML algorithms, HDBSCAN and OPTICS require numerical feature vectors as input, making feature encoding an essential component. These biological feature descriptors transform variable-length DNA sequences into fixed-length numerical vectors of substantially lower dimensionality, providing biologically meaningful information for taxonomic discrimination and enabling the application of clustering algorithms. All descriptors were computed as frequency-based metrics normalized by sequence length, such that the resulting feature values represent compositional proportions rather than raw counts, ensuring comparability across sequences of varying length. For instance, nucleic acid composition (NAC) represents the relative frequencies of the 4 nucleotides (A, T, G, and C), and a higher NAC value for a given nucleotide indicates a greater proportion of that base within the sequence. Conversely, composition of k-spaced nucleic acid pairs (CKSNAP) additionally captures the frequencies of nucleotide pairs separated by defined gap lengths, thereby incorporating local sequence-order information [57]. In this study, we exploited 19 descriptors that were used extensively in previous studies [57–59]. These descriptors represented 4 major descriptor groups (i.e., NAC, electron–ion interaction pseudopotentials, autocorrelation and cross-covariance, and pseudo NAC) and comprised 694 biological features extracted from the metabarcoding sequences using the stand-alone toolkit iLearn [57]. A detailed description of these features is presented in Table S2. All feature vectors were further standardized using *z*-score normalization to ensure that features with different numerical ranges contributed equally to the distance metric.

### UML model and performance evaluation metrics

HDBSCAN version 0.8.33 [60] and OPTICS [51] from scikit-learn version 1.0.2 [61] were used to cluster metabarcoding data using biological features (Supplementary Materials and Methods S2-1). These density-based methods identify clusters with varying densities and shapes. HDBSCAN constructs a cluster hierarchy and extracts the most stable clusters without requiring the final dendrogram to be cut at a single point, separating it from other hierarchical techniques [62]. Similarly, OPTICS generates a reachability plot to visualize the density-based structure of the dataset, making it effective for identifying clusters in data with nonuniform densities without requiring a predefined density threshold [51]. The clustering results were visualized as a principal component analysis (PCA) plot using the PCA function from scikit-learn version 1.0.2 [61].

Three complementary indices were used to evaluate clustering quality (Supplementary Materials and Methods S2-1). The silhouette score measures intracluster cohesion and intercluster separation and ranges from −1 to 1, with higher scores indicating that objects are better matched to their own clusters and poorly matched to neighboring clusters, suggesting a good clustering configuration [63]. The Davies–Bouldin Index (DBI; [64]) assesses cluster compactness relative to intercluster distances, with lower values indicating better separated clusters. The Calinski–Harabasz Index (CHI; [65]) evaluates intercluster separability as the ratio of between-cluster to within-cluster dispersion, with higher values indicating more distinct cluster structures.

### Taxonomic assignment and comparative analysis

To ensure a consistent comparison among methods, the UML-based methods, OTU clustering, and ASV analysis were applied to the same 6 mock community datasets. For OTU and UML-based methods, an identical preprocessing pipeline was applied before method-specific clustering steps, as described in the “Preprocessing metabarcoding sequences” section and Supplementary Materials and Methods S2-1. For ASV analysis, preprocessing was performed using the DADA2 pipeline (Supplementary Materials and Methods S2-2).

Taxonomic assignment was performed consistently across all 3 methods using the consensus-BLAST function with the q2-feature-classifier plugin of QIIME2 [66], applying the default similarity threshold of 80% (--p-perc-identity = 0.8; Supplementary Materials and Methods S3). For the UML-based methods, reads from each cluster were extracted using dplyr 1.1.1 in R version 4.2.3. Singleton clusters were excluded prior to assignment. The reference database was MitoFish [67] for 12S rRNA sequences, BOLD Systems [68] for COI sequences, and SILVA [69] for 18S rRNA sequences.

Processed sequences from all methods were normalized and aggregated into taxonomic profiles at the order, family, genus, and species levels. Sequences not assigned to a valid taxon, including those not classified as Actinopteri for 12S rRNA, were excluded from downstream analyses.

### Comparison of the UML, OTU, and ASV methods

Based on the number of reads assigned to each taxon, within-sample taxon-wise relative abundance was calculated at the order, family, genus, and species levels using R version 4.2.3 and the phyloseq package [70]. To evaluate the similarity of the analytical methods, Procrustes analysis was performed using the Procrustes function in the Vegan package version 2.6.4 [71] based on relative abundance data. The statistical significance of pairwise comparisons was assessed using permutation tests (999 permutations; for datasets with few samples, the maximum number of permutations under complete enumeration was used). The taxa detected by the UML, OTU, and ASV methods were visualized as UpSet plots created utilizing UpSetR version 1.4.0 [72] in R version 4.2.3. During this process, taxa such as “sp.” and “uncultured”, which are not appropriate for the given taxonomic level, were excluded.

To evaluate the biological coherence of the UML-derived clusters, within-cluster taxonomic consistency was assessed at the order, family, genus, and species levels. For each cluster, consistency was calculated as the proportion of reads assigned to the most frequent taxon at the corresponding taxonomic rank, and mean consistency was subsequently calculated across clusters. The stability of the dominant taxonomic assignment was assessed using bootstrap resampling with 100 replicates. For each genetic marker, the reported values represent the means across the 2 mock community datasets. The percentage of clusters showing within-cluster consistency of at least 90% was also calculated. An equivalent read-level analysis was not conducted for OTU and ASV outputs because read-to-feature membership information was not available from the retained outputs of these workflows.

To evaluate the relative utility of species detection for each method, the numbers of species identified using UML, OTU, and ASV analysis data were compared. The sensitivity, precision, and F1 score of the metabarcoding analyses were calculated with modifications based on a previously reported method [3].

In this study, actual positives were defined as species in the mock community that were identified by any metabarcoding method. This definition reflects the assumption that species undetected using any method might fall outside the current detectable range of metabarcoding approaches. Predicted positives were defined as species detected using a given metabarcoding method, whereas predicted negatives were species not detected using the method. True positives (TPs) were species included in the mock community and correctly detected as positives using the metabarcoding method. False positives (FPs) were species not included in the mock community but incorrectly detected as positives. False negatives (FNs) were species included in the mock community that were part of the actual positives but not detected as positives. Accordingly, sensitivity was calculated as the proportion of TPs among these actual positives (Eq. 1). Precision, or positive predictive value, reflects the proportion of TPs among all predicted positives (Eq. 2). The performance metrics were evaluated to compare the effectiveness of the UML, OTU, and ASV approaches in detecting target species. By combining sensitivity and precision, the F1 score was calculated to provide a balanced assessment of detection accuracy (Eq. 3).

$$\begin{array}{c} \text{Sensitivity}=\\ \frac{\text{Number of}\;\mathrm{TPs}}{\text{Number of positives in the mock community}\;\left(\mathrm{TPs}+\mathrm{FNs}\right)} \end{array}$$ (1)

$$\begin{array}{c} \text{Precision}=\\ \frac{\text{Number of}\;\mathrm{TPs}}{\text{Number of positives detected}\;\mathrm{by}\;\text{the method}\;\left(\mathrm{TPs}+\mathrm{FPs}\right)} \end{array}$$ (2)

$$F1\;\text{score}=\frac{2\times \left(\text{Precision}\times \text{Sensitivity}\right)}{\text{Precision}+\text{Sensitivity}}$$ (3)

## Results and Discussion

### Development of the UML-based methods

From all 6 datasets, 33,652,032 reads (Study 1) and 913,455 reads (Study 2) were collected for the 12S rRNA marker, 401,031 and 368,176 reads were collected for the COI marker, and 2,198,735 and 953,564 reads were collected for the 18S rRNA marker (Tables S3 to S8). All collected mock community metabarcoding data were preprocessed for the UML-based methods. After adapter trimming, pair-merged sequences were subjected to dereplication and chimera sequence filtering, resulting in 30,424,820 and 725,341 sequences (90.4% and 79.4% of raw reads, respectively) for the 12S rRNA datasets, 379,509 and 187,159 sequences (94.6% and 50.8%, respectively) for the COI datasets, and 1,926,388 and 432,980 sequences (87.6% and 45.4%, respectively) for the 18S rRNA datasets (Tables S3 to S8). These processed sequences were then used for the UML-based methods.

**Fig. 2.**
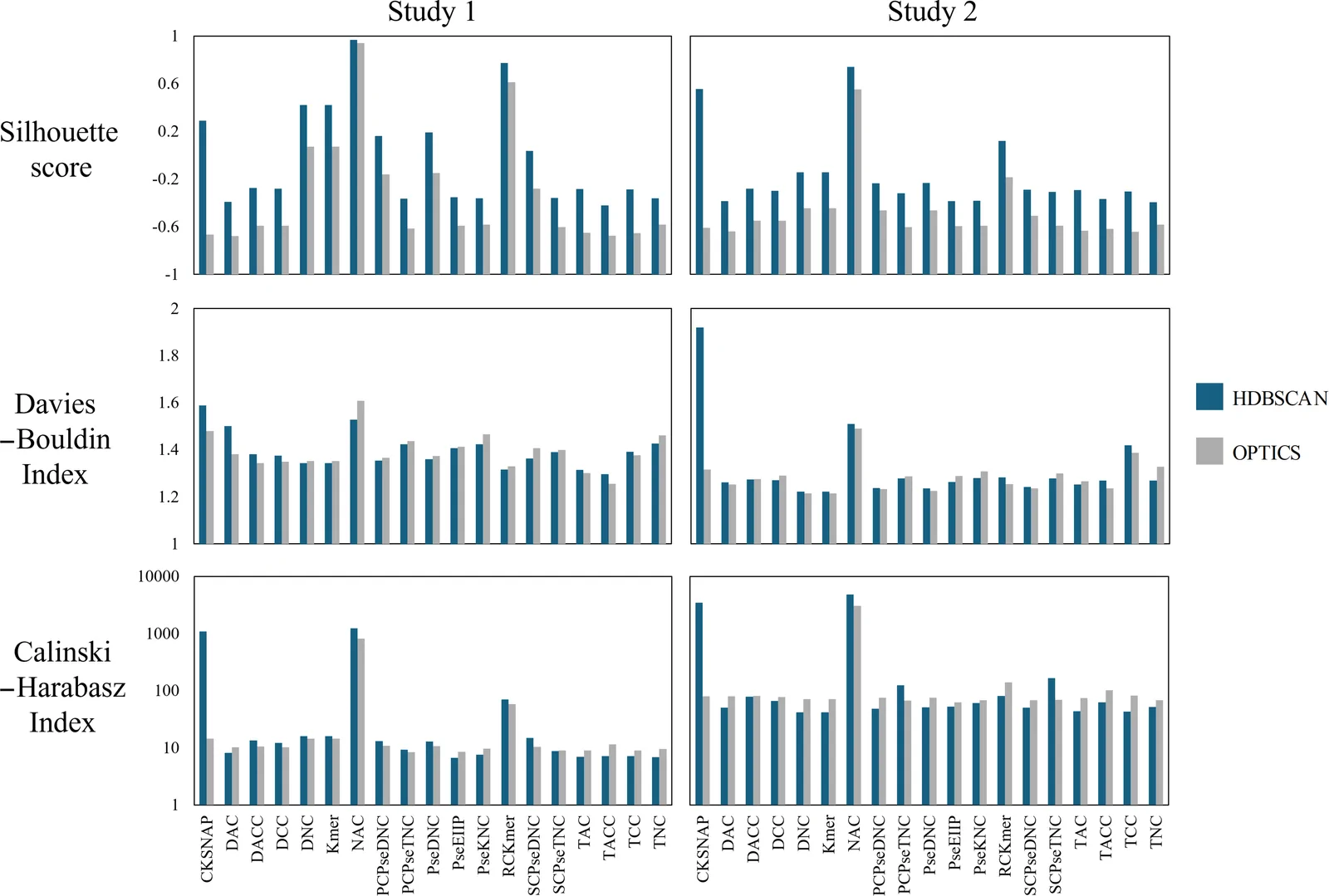
Clustering performance metrics for HDBSCAN and OPTICS applied to the 12S rRNA mock community metabarcoding dataset. The *X*-axis presents the 19 biological features used for clustering, whereas the *Y*-axis presents the 3 clustering performance evaluation metrics. Blue bars represent the results for HDBSCAN, and gray bars represent the results for OPTICS. Each bar corresponds to the performance metric for a specific biological feature. NAC, nucleic acid composition; DNC, dinucleotide composition; TNC, trinucleotide composition; CKSNAP, k-spaced nucleic acid pairs; Kmer, basic k-mer composition; RCKmer, reverse-complement k-mer composition; PseEIIP, electron–ion interaction pseudopotential of trinucleotides; DAC, dinucleotide-based auto covariance; DCC, dinucleotide-based cross covariance; DACC, dinucleotide-based auto cross covariance; TAC, trinucleotide-based auto covariance; TCC, trinucleotide-based cross covariance; TACC, trinucleotide-based auto cross covariance; PseDNC, pseudo dinucleotide composition; PseKNC, pseudo k-tupler composition; PCPseDNC, parallel correlation pseudo dinucleotide composition; PCPseTNC, parallel correlation pseudo trinucleotide composition; SCPseDNC, series correlation pseudo dinucleotide composition; SCPseTNC, series correlation pseudo trinucleotide composition

**Fig. 3.**
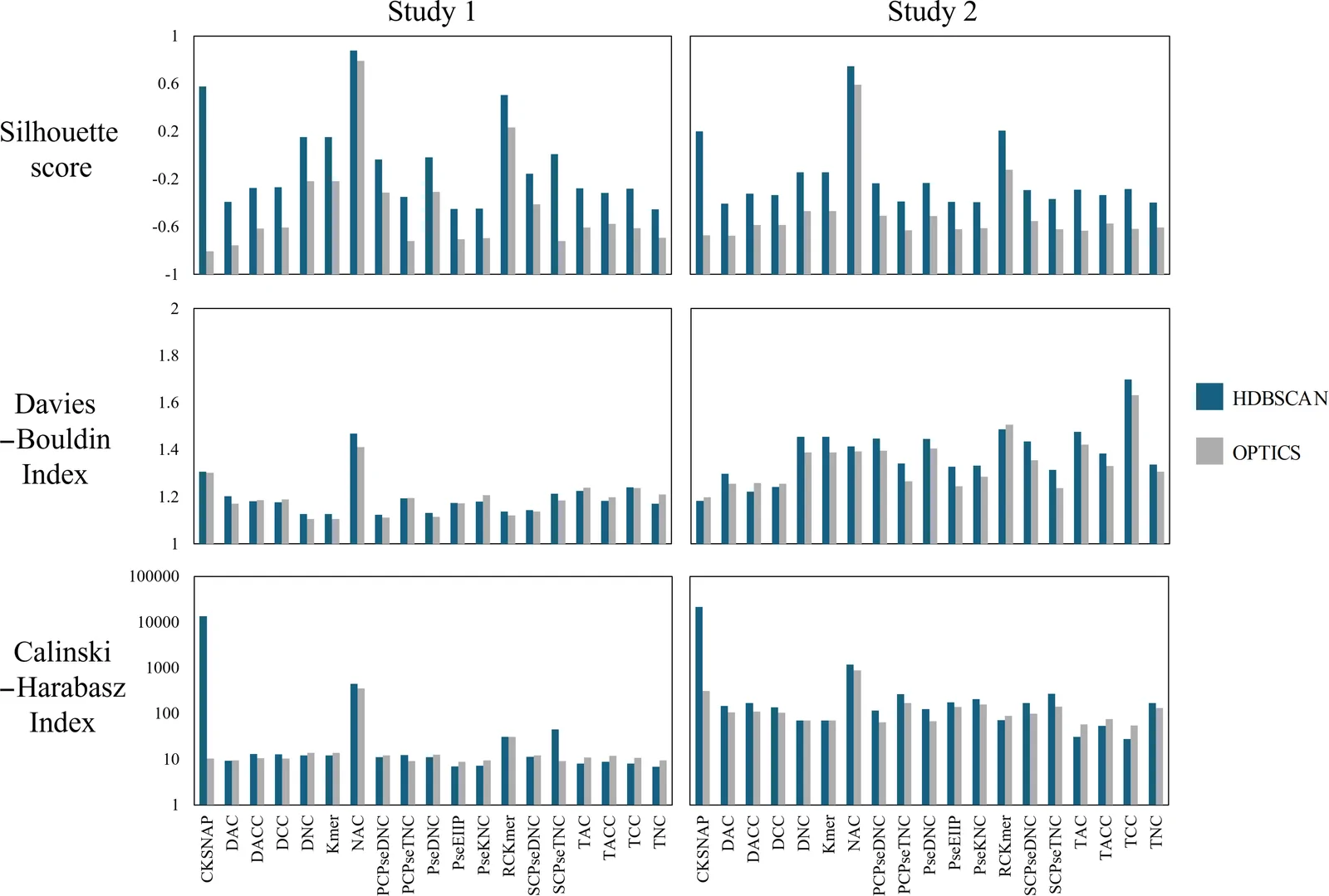
Clustering performance metrics for HDBSCAN and OPTICS applied to the COI mock community metabarcoding dataset. The *X*-axis presents the 19 biological features used for clustering, and the *Y*-axis presents the 3 clustering performance evaluation metrics. Blue bars represent the results for HDBSCAN, and gray bars represent the results for OPTICS. Each bar corresponds to the performance metric for a specific biological feature. NAC, nucleic acid composition; DNC, dinucleotide composition; TNC, trinucleotide composition; CKSNAP, k-spaced nucleic acid pairs; Kmer, basic k-mer composition; RCKmer, reverse-complement k-mer composition; PseEIIP, electron–ion interaction pseudopotential of trinucleotides; DAC, dinucleotide-based auto covariance; DCC, dinucleotide-based cross covariance; DACC, dinucleotide-based auto cross covariance; TAC, trinucleotide-based auto covariance; TCC, trinucleotide-based cross covariance; TACC, trinucleotide-based auto cross covariance; PseDNC, pseudo dinucleotide composition; PseKNC, pseudo k-tupler composition; PCPseDNC, parallel correlation pseudo dinucleotide composition; PCPseTNC, parallel correlation pseudo trinucleotide composition; SCPseDNC, series correlation pseudo dinucleotide composition; SCPseTNC, series correlation pseudo trinucleotide composition.

**Fig. 4.**
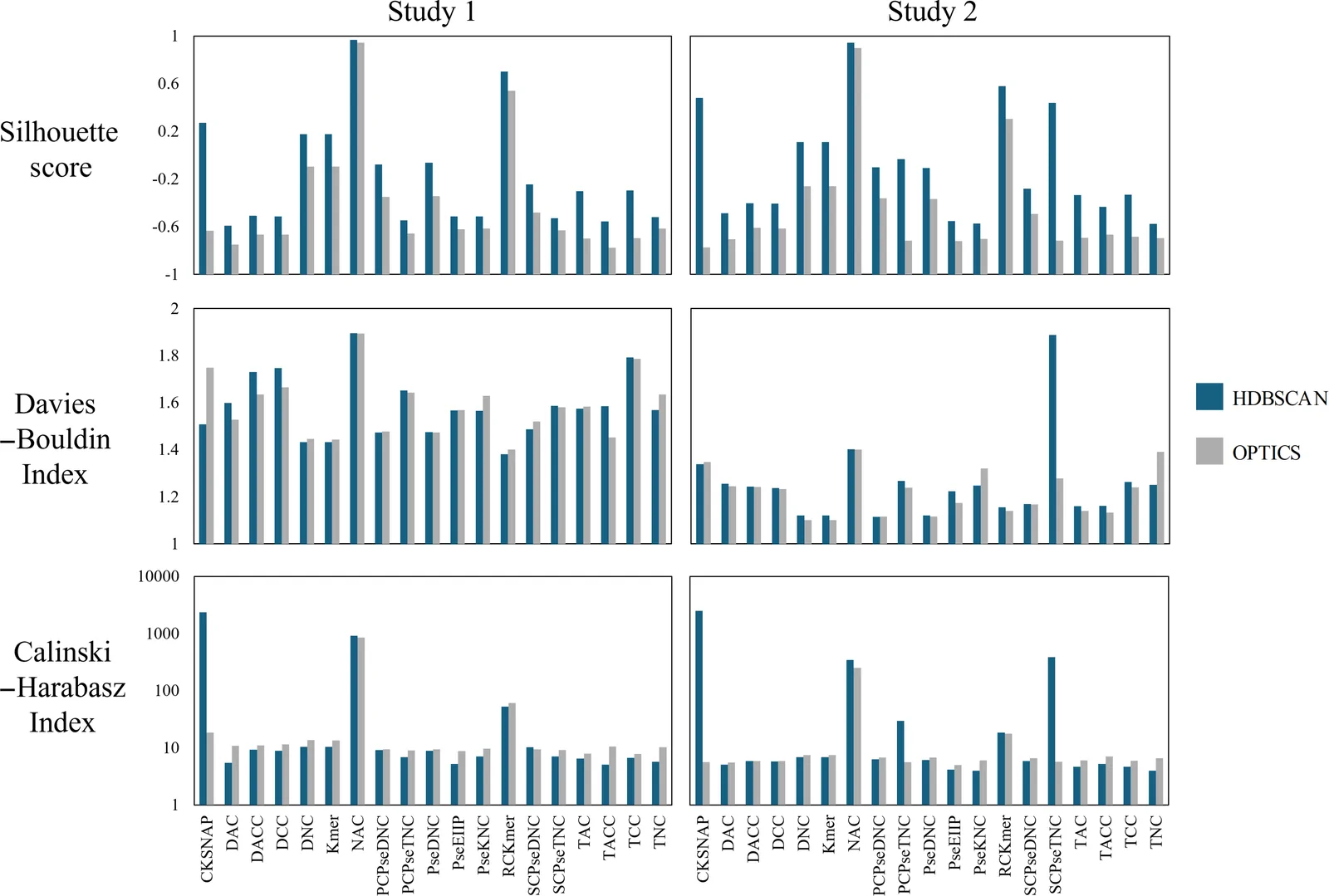
Clustering performance metrics for HDBSCAN and OPTICS applied to the 18S rRNA mock community metabarcoding dataset. The *X*-axis presents the 19 biological features used for clustering, and the *Y*-axis presents the 3 clustering performance evaluation metrics. Blue bars represent the results for HDBSCAN, and gray bars represent the results for OPTICS. Each bar corresponds to the performance metric for a specific biological feature. NAC, nucleic acid composition; DNC, dinucleotide composition; TNC, trinucleotide composition; CKSNAP, k-spaced nucleic acid pairs; Kmer, basic k-mer composition; RCKmer, reverse-complement k-mer composition; PseEIIP, electron–ion interaction pseudopotential of trinucleotides; DAC, dinucleotide-based auto covariance; DCC, dinucleotide-based cross covariance; DACC, dinucleotide-based auto cross covariance; TAC, trinucleotide-based auto covariance; TCC, trinucleotide-based cross covariance; TACC, trinucleotide-based auto cross covariance; PseDNC, pseudo dinucleotide composition; PseKNC, pseudo k-tupler composition; PCPseDNC, parallel correlation pseudo dinucleotide composition; PCPseTNC, parallel correlation pseudo trinucleotide composition; SCPseDNC, series correlation pseudo dinucleotide composition; SCPseTNC, series correlation pseudo trinucleotide composition.

To identify the most suitable biological features for the UML-based method, HDBSCAN and OPTICS were applied to biological features extracted from the 12S rRNA, COI, and 18S rRNA mock community metabarcoding datasets (Figs. 2 to 4, Figs. S1 to S3, and Tables S9 to S15). Clustering performance was assessed using the silhouette score, DBI, CHI, and number of clusters.

#### Clustering analysis of 12S rRNA metabarcoding data

For HDBSCAN in Study 1, NAC achieved the highest CHI (1,224.720) and silhouette score (0.968), indicating well-separated clusters (Table S9, Fig. 2, and Fig. S1). In Study 2, the silhouette score of CKSNAP increased to 0.556. NAC maintained strong performance with a high CHI (69.426). Conversely, TAC and DNC exhibited negative silhouette scores and lower CHIs (Table S10, Fig. 2, and Fig. S1).

Using OPTICS in Study 1, NAC remained robust, with a high CHI (816.724) and relatively compact clusters. CKSNAP produced a moderate number of clusters but exhibited poor separation (silhouette score = −0.666; Table S9, Fig. 2, and Fig. S1). In Study 2, OPTICS again indicated the superior performance of NAC, with NAC forming the most distinct clusters alongside a manageable cluster count, whereas DAC and TNC generated more dispersed clusters (Table S10, Fig. 2, and Fig. S1).

Overall, NAC was the most reliable feature for clustering the 12S rRNA dataset. Conversely, CKSNAP exhibited inconsistent performance. RCKmer and SCPseDNC displayed moderate performance but remained secondary options.

#### Clustering analysis of COI metabarcoding data

The clustering of various biological features for the COI mock community metabarcoding dataset was performed using HDBSCAN and OPTICS (Fig. 3, Fig. S2, and Tables S11 and S12). For the COI dataset, NAC consistently demonstrated superior performance across all features. In Study 1, NAC achieved the highest silhouette score (0.876), forming 2,644 unique clusters and 5,765 total clusters. CKSNAP also exhibited moderate performance, achieving a silhouette score of 0.575. In Study 2 with HDBSCAN, NAC maintained its leading performance with a silhouette score of 0.746 (Fig. 3). It formed 6,528 unique clusters and 12,156 total clusters. The OPTICS results for the COI dataset revealed similar trends. In Study 1, NAC emerged as the most robust feature, achieving a silhouette score of 0.790 and a CHI of 349 (Fig. 3). It formed 5,729 unique clusters and 7,576 total clusters. In Study 2 using OPTICS, NAC outperformed all other features, producing cohesive clusters with minimal overlap (Fig. 3). NAC achieved a silhouette score of 0.590 and a DBI of 1.393, forming 14,329 unique clusters and 16,982 total clusters (Fig. 3 and Table S12). The results across both studies and clustering algorithms indicated that although CKSNAP had high values in some cases, NAC was generally the most reliable feature for the COI dataset.

#### Clustering analysis of 18S rRNA metabarcoding data

The clustering performance of various biological features for the 18S rRNA mock community metabarcoding dataset was assessed using HDBSCAN and OPTICS (Fig. 4, Fig. S3, and Tables S13 and S14).

For HDBSCAN, NAC consistently outperformed other features in Study 1. NAC achieved the highest silhouette score (0.968, Table S13), and CKSNAP achieved the highest CHI (2,335.304). NAC and CKSNAP formed 3,505 and 503 unique clusters, respectively, and 9,199 and 580 total clusters, respectively. In Study 2 with HDBSCAN, NAC maintained its superior clustering quality, achieving a silhouette score of 0.943, whereas CKSNAP achieved the highest CHI of 17,724.140 (Fig. 4). They produced 1,318 and 417 unique clusters and 3,378 and 467 total clusters, respectively.

The OPTICS results further highlighted the superior performance of NAC. In Study 1, NAC achieved a silhouette score of 0.943, forming cohesive and distinct clusters (Fig. 4). In Study 2 using OPTICS, NAC retained its superior performance with a silhouette score of 0.898. Its clusters were cohesive and well-separated, with a manageable total of 4,419 clusters (Table S14).

These results, similar to those observed in the COI dataset, indicate that although CKSNAP achieved high values in some cases, NAC was generally the most reliable feature for clustering the 18S rRNA dataset.

#### Evaluation of clustering performance based on biological features and algorithms

In this study, we assessed the clustering performance of 19 biological features extracted from metabarcoding datasets (12S rRNA, COI, and 18S rRNA) using HDBSCAN and OPTICS. Among the evaluated biological features, NAC demonstrated consistent superiority, achieving the highest silhouette scores across all datasets and clustering algorithms. The discriminatory power of NAC likely reflects taxon-specific base compositional biases that arise from evolutionary processes such as mutational pressure, codon usage bias, and selective constraints, which are preserved even in short barcode regions [73]. This low-dimensional yet biologically informative representation enables the formation of compact, well-separated clusters. However, this interpretation remains provisional. The datasets analyzed in this study comprised taxa with relatively broad evolutionary divergence, which might favor low-dimensional compositional descriptors such as NAC. Consequently, the performance of NAC might decline in datasets containing closely related species, shallow sequence divergence, or extensive intraspecific polymorphism, in which compositional differences among taxa become less distinct. The robustness of NAC across diverse taxonomic and ecological conditions therefore warrants further evaluation.

Conversely, CKSNAP exhibited the highest CHI in the COI and 18S rRNA datasets. By encoding the frequencies of nucleotide pairs separated by k intervening bases, CKSNAP captures local sequence context beyond simple base composition [74] and provides additional discriminatory information in taxonomically complex datasets. However, the curse of dimensionality might partially explain the lower silhouette scores of CKSNAP: as dimensionality increases, distances between points tend to converge, reducing apparent cluster separability [75]. The contrasting performance of NAC and CKSNAP across datasets could reflect differences in amplicon length and taxonomic scope. In the 12S rRNA dataset, in which amplicons were relatively short, the low-dimensional representation of NAC might provide a sufficient compositional signal for cluster separation. Contrarily, for the COI and 18S rRNA datasets, which target more taxonomically diverse groups and produce longer amplicons, the higher-order sequence context captured by CKSNAP might offer additional discriminatory information that simple nucleotide composition alone cannot provide.

HDBSCAN consistently outperformed OPTICS in producing compact and well-separated clusters, as reflected by its higher silhouette scores across most datasets and features. Its automatic noise handling, which assigns low-density points as noise rather than forcing them into clusters [48], was particularly beneficial in metabarcoding datasets in which outliers and sparse data points can skew clustering results, and its performance remained stable across datasets with varying cluster shapes and densities. Furthermore, the interaction between feature dimensionality and clustering algorithm influenced optimal feature selection: HDBSCAN exhibited superior performance with CKSNAP in the COI and 18S rRNA datasets, suggesting that its hierarchical density estimation can effectively exploit high-dimensional feature spaces. Conversely, OPTICS consistently favored NAC across these datasets, likely because of the reduced stability of density estimation in high-dimensional spaces, making low-dimensional features more reliable for this algorithm.

Meanwhile, OPTICS demonstrated greater flexibility in exploring datasets with clusters of variable density but generally produced lower silhouette scores than HDBSCAN. Despite its advantages in identifying hierarchical relationships and overlapping clusters, OPTICS struggled to form compact clusters, and its greater computational complexity and sensitivity to parameter settings make it less practical for large-scale metabarcoding analyses than HDBSCAN (Tables S9 to S14).

### Comparison of the UML-based methods, OTU clustering, and ASV analysis

In developing the UML-based method, evaluation using the silhouette score, DBI, and CHI confirmed the optimal performance of the UML-based methods using NAC or CKSNAP descriptors (Table S15). To compare taxon-wise relative abundance among analytical approaches, results obtained using the best-performing UML descriptor were compared with those from OTU clustering and ASV analysis. Detailed preprocessing steps and taxon-wise relative abundance results for the OTU and ASV analyses are provided in Tables S16 to S21 and Figs. S4 to S9.

#### Comparison of relative abundance

The analysis of species-level relative abundance across the 12S rRNA, COI, and 18S rRNA datasets revealed varying degrees of similarity and divergence among the UML (HDBSCAN and OPTICS), OTU, and ASV methods. Overall, HDBSCAN generally produced relative-abundance patterns that were more consistent with those obtained by OTU clustering and ASV analysis than those produced by OPTICS. In the 12S rRNA datasets, HDBSCAN exhibited abundance profiles broadly similar to those of OTU and ASV, whereas OPTICS detected additional variation among low-abundance taxa. A similar pattern was observed using the COI datasets, although OPTICS exhibited substantially greater divergence from the other methods in Study 2. For the 18S rRNA datasets, HDBSCAN again displayed relative abundance patterns comparable to those of OTU and ASV, whereas differences among the methods were minimal in Study 2. These findings suggest that HDBSCAN provides more consistent relative abundance estimates across diverse metabarcoding datasets, whereas OPTICS might be more sensitive to low-abundance taxa. Detailed taxon-wise relative abundance profiles are provided in Figs. S4 to S9.

Procrustes analysis was performed to statistically compare relative abundance patterns among methods (Figs. S10 to S12). Overall, HDBSCAN, OTU, and ASV displayed high structural congruence across most datasets and taxonomic levels, whereas OPTICS exhibited greater divergence in several datasets. This pattern was particularly evident in COI Study 2, in which OPTICS differed more strongly from the other methods across taxonomic levels, whereas HDBSCAN, OTU, and ASV remained highly similar. In the 18S rRNA datasets, all methods exhibited strong congruence in Study 1, but greater divergence was observed at finer taxonomic levels in Study 2. These results support the relative consistency of HDBSCAN with conventional OTU and ASV approaches in estimating taxon-wise abundance patterns. Detailed Procrustes results are provided in Figs. S10 to S12, and the corresponding Procrustes correlation coefficients, *M*^2^ values, and exact *P* values are provided in Table S22.

The Procrustes analysis results across the 12S rRNA, COI, and 18S rRNA datasets revealed varying degrees of similarity among the clustering methods. At finer taxonomic levels, such as genus and species, HDBSCAN, OTU, and ASV consistently displayed structural similarity in abundance patterns. By contrast, OPTICS exhibited moderate divergence, particularly at higher taxonomic levels (order and family) and in datasets with greater complexity (e.g., COI and 18S rRNA Study 2). This divergence could reflect the greater sensitivity of OPTICS to variations in dataset structure and density distribution observed in 18S rRNA Study 1, in which *M*^2^ = 0.00 across all taxonomic levels, highlighting the homogeneity of this dataset and allowing all methods to produce structurally congruent abundance patterns. Conversely, the increasing divergence in 18S rRNA Study 2 and COI Study 2 emphasizes the need for method selection tailored to the dataset’s complexity and taxonomic resolution demands. In summary, HDBSCAN, OTU, and ASV were consistently robust methods for taxonomic clustering across diverse datasets. OPTICS, although useful, requires parameter tuning and validation to achieve comparable performance, particularly for complex datasets. Future studies should explore hybrid approaches and adaptive parameterization to optimize clustering outcomes across varied dataset characteristics.

#### Comparison of detected species

**Fig. 5.**
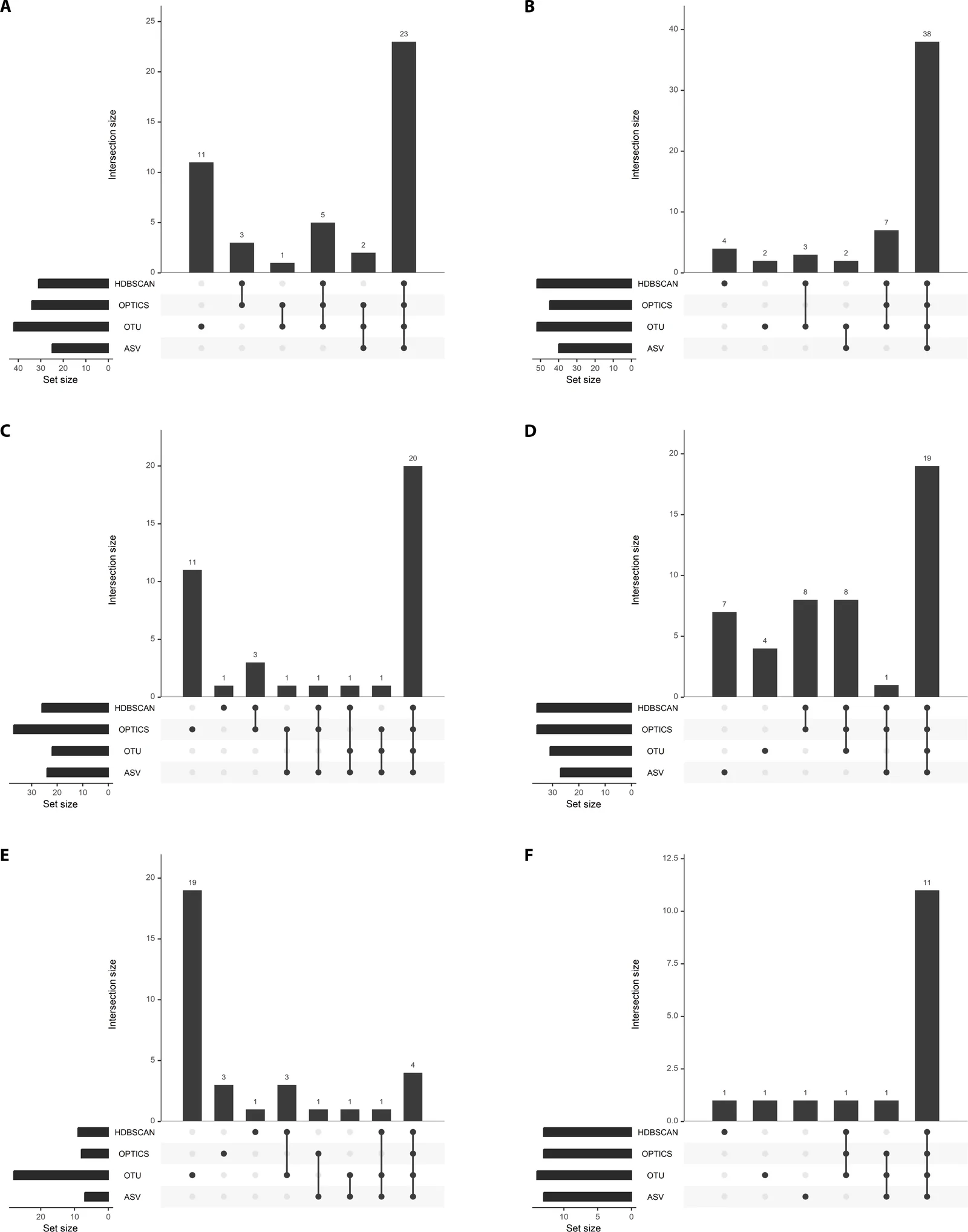
UpSet plots visualizing the overlap of detected species among the HDBSCAN, OPTICS, OTU, and ASV methods across 12S rRNA, COI, and 18S rRNA datasets. (A) 12S rRNA Study 1. (B) 12S rRNA Study 2. (C) COI Study 1. (D) COI Study 2. (E) 18S rRNA Study 1. (F) 18S rRNA Study 2. Each plot illustrates the unique and shared species detected by the methods, with horizontal bars representing the total number of species identified by each method and vertical bars indicating the size of intersections among the methods.

**Fig. 6.**
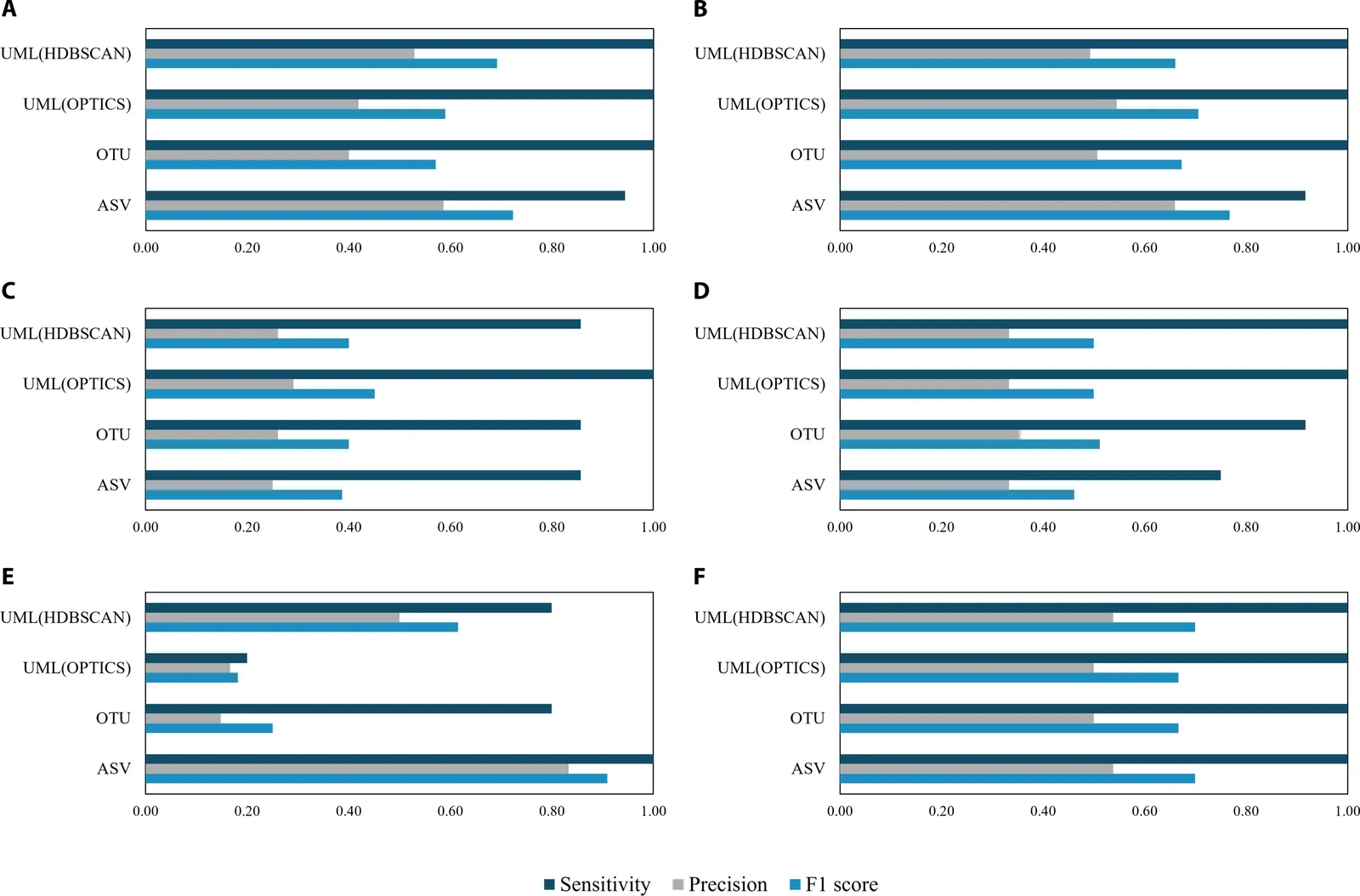
Bar charts comparing the performance metrics (sensitivity, precision, and F1 score) of HDBSCAN, OPTICS, OTU, and ASV methods across 12S rRNA, COI, and 18S rRNA datasets. (A) 12S rRNA Study 1. (B) 12S rRNA Study 2. (C) COI Study 1. (D) COI Study 2. (E) 18S rRNA Study 1. (F) 18S rRNA Study 2. Each bar represents a performance metric for each method, providing a comprehensive comparison of their sensitivity, precision, and F1 scores across different datasets and studies.

To understand the differences in the species detected by each method, we analyzed the species identified by HDBSCAN, OPTICS, OTU, and ASV in relation to the species composition of the mock community. The results, as summarized in Table 1 (performance metrics presented in Figs. 5 and 6), indicated that HDBSCAN maintained sensitivity equal to or higher than that of OTU clustering in all 6 datasets and achieved equal or higher precision in 4 of the 6 datasets, although the relative performance of the 4 methods varied among datasets.

**Table 1.** Detected species among UML-based methods (HDBSCAN and OPTICS), OTU clustering (OTU), and ASV analysis (ASV)

Genetic marker	Study	Methods				
		Mock community	HDBSCAN	OPTICS	OTU	ASV
**12S rRNA**	Study 1	21	18	18	18	17
	Study 2	40	36	36	36	33
**COI**	Study 1	26	6	7	6	6
	Study 2	24	12	12	11	9
**18S rRNA**	Study 1	7	4	1	4	5
	Study 2	12	7	7	7	7

In the 12S rRNA datasets, HDBSCAN consistently achieved high sensitivity (1.00) across both studies, as did OTU and OPTICS. In Study 1, HDBSCAN attained an F1 score of 0.69 and precision of 0.53, outperforming OTU (0.57 and 0.40, respectively) and OPTICS (0.59 and 0.42, respectively; Table 1 and Figs. 5A and 6A). ASV achieved high precision (0.59) but lower sensitivity (0.94) and an F1 score of 0.72, indicating a higher FN rate than HDBSCAN. In Study 2, ASV exhibited the highest precision (0.66) and F1 score (0.77) among the methods, but its sensitivity (0.92) was noticeably lower, indicating its tendency to miss some TPs and generate more FNs. OTU demonstrated perfect sensitivity (1.00), a precision of 0.51, and an F1 score of 0.67. Similarly, OPTICS also achieved perfect sensitivity (1.00), alongside a slightly higher precision (0.55) and a higher F1 score (0.71), suggesting a better balance between precision and recall than OTU. HDBSCAN achieved perfect sensitivity (1.00) but the lowest precision (0.49) and F1 score (0.66) among the 4 methods in this dataset (Figs. 5B and 6B).

In the COI datasets, greater variability was observed among the methods, particularly in Study 1. HDBSCAN had an F1 score of 0.40 and a precision of 0.26, performing similarly to OTU (0.40 and 0.26, respectively). ASV demonstrated a slightly lower precision (0.25) but achieved a similar F1 score (0.39). OPTICS outperformed the other methods, achieving the highest F1 score (0.45) and precision (0.29), alongside perfect sensitivity (1.00), indicating that OPTICS can provide a more balanced approach under such conditions (Figs. 5C and 6C). In Study 2, HDBSCAN showed improved performance, achieving an F1 score of 0.50, equivalent to that of OPTICS, and a precision of 0.33, matching that of ASV but slightly lower than that of OTU (0.35). Both HDBSCAN and OPTICS achieved perfect sensitivity (1.00), whereas ASV and OTU had lower sensitivity (0.75 and 0.92, respectively). These results suggest that HDBSCAN and OPTICS are better equipped to detect TPs in moderately complex datasets without sacrificing sensitivity (Figs. 5D and 6D).

In the 18S rRNA datasets, HDBSCAN achieved higher precision and F1 scores than OTU and OPTICS in both studies while maintaining comparable or higher sensitivity. In Study 1, HDBSCAN achieved a precision of 0.50 and a sensitivity of 0.80, resulting in an F1 score of 0.62, reflecting a substantial improvement over OTU (F1 score= 0.25, precision= 0.15, sensitivity= 0.80) and OPTICS (F1 score= 0.18, precision= 0.17, sensitivity= 0.20). ASV had the highest F1 score (0.91), precision (0.83), and sensitivity (1.00), indicating exceptional performance. However, HDBSCAN offered a stronger balance between sensitivity and precision than OTU and OPTICS, making it more robust for moderately complex datasets (Figs. 5E and 6E). In Study 2, HDBSCAN displayed consistent performance, achieving an F1 score of 0.70, a precision of 0.54, and a sensitivity of 1.00, matching ASV’s performance across all metrics. OTU and OPTICS also achieved strong results, with an F1 score of 0.67 and a precision of 0.50, alongside perfect sensitivity (1.00). These results demonstrate that HDBSCAN and ASV balance sensitivity and precision, whereas OTU and OPTICS had comparable performance but slightly lower precision and F1 scores (Table 1 and Figs. 5F and 6F).

Across the datasets, the relative performance of HDBSCAN, OPTICS, OTU, and ASV varied among datasets. HDBSCAN generally maintained high sensitivity while achieving reasonable precision in several datasets. ASV exhibited higher precision in some datasets but showed reduced sensitivity in others. OTU generally maintained high sensitivity but exhibited lower precision in several datasets, whereas OPTICS showed more variable performance across datasets. By achieving high sensitivity while maintaining reasonable precision in several datasets, HDBSCAN may provide a useful option when comprehensive species detection is prioritized. Moreover, the comparative results revealed distinct dataset-specific trade-offs among the methods. For example, OPTICS showed low sensitivity in 18S rRNA Study 1 (0.20), OTU clustering showed low precision in the same dataset (0.15), and ASV analysis showed reduced sensitivity in COI Study 2 (0.75). In contrast, HDBSCAN maintained relatively balanced sensitivity and precision across the evaluated mock community datasets. These results suggest that HDBSCAN may provide a comparatively stable option across different datasets, although its robustness under broader environmental conditions remains to be validated.

To further evaluate whether the resulting clusters correspond to biologically meaningful units, we assessed the taxonomic homogeneity of each UML-based cluster, which was quantified as the proportion of reads within a cluster assigned to the most frequent taxon at the order, family, genus, and species levels, with the stability of the dominant assignment confirmed by bootstrap resampling (Table S23). Within-cluster consistency was high at higher taxonomic ranks, reaching approximately 98% at the order level and high values at the family and genus levels in most datasets, supporting the presence of substantial taxonomic structure within the UML-derived clusters. Consistency decreased toward the species level and varied among the datasets. Because per-read cluster membership cannot be recovered for OTU and ASV outputs, an equivalent read-level comparison with these methods was not feasible.

The findings of this study underscore the critical role of clustering methods in taxonomic profiling and their effects on the detection of species in complex datasets. Among the tested methods, HDBSCAN showed a favorable balance between sensitivity and precision in several datasets, generally maintaining high sensitivity while achieving reasonable precision. However, its relative performance varied among datasets, indicating that its advantages depend on dataset characteristics and analytical objectives.

ASV generally showed competitive precision, although its relative performance varied among datasets. For example, in COI Study 2, ASV had lower sensitivity (0.75), indicating a tendency to miss some TPs. Despite its generally competitive precision, ASV methods might require additional filtering or validation steps to ensure comprehensive species detection, particularly for datasets with high diversity of low-abundance taxa [76]. Conversely, OTU clustering demonstrated consistently high sensitivity in most datasets, but its lower precision (e.g., 0.40 in 12S rRNA Study 1 and 0.15 in 18S rRNA Study 1) indicates a higher propensity for FPs. This limitation is particularly evident in datasets with sparse or uneven taxonomic representation, in which OTU methods might generate erroneous detections. These findings suggest that although OTU clustering reliably captures a broad range of TPs, it might require additional filtering to minimize FPs [77].

The differences in performance among the methods highlight the importance of selecting a clustering approach according to the objectives of the study. Across most datasets, HDBSCAN maintained sensitivity comparable to or higher than that of OTU clustering while achieving comparable or improved precision and F1 scores. This indicates that HDBSCAN can provide the comprehensive detection commonly sought from OTU clustering while markedly reducing FPs (e.g., precision 0.50 vs. 0.15 in 18S rRNA Study 1 at equal sensitivity). The principal trade-off therefore lies between HDBSCAN and ASV analysis: ASV attains higher precision and, in several datasets, higher F1 scores, but it can miss true species (e.g., sensitivity of 0.75 in COI Study 2). Based on these results, HDBSCAN may be particularly useful when comprehensive detection is prioritized and FNs are considered more consequential than a moderate number of FPs, such as in exploratory presence/absence biodiversity inventories, because it maintained high sensitivity while retaining reasonable precision in several datasets. More broadly, UML-based methods may also provide an alternative when a clustering workflow that does not require a predefined sequence-similarity threshold or a platform- and run-specific error model is preferred. When maximizing precision is the primary objective, ASV analysis may be preferable, whereas OTU clustering might be adequate when simple threshold-based grouping is sufficient. These differences support the complementary use of methods with distinct analytical strengths, consistent with previous research showing that different analytical methods can capture different aspects of complex biological data and provide complementary information [78]. However, the suitability of UML-based methods for detecting rare, invasive, or endangered taxa requires further validation using environmental datasets and appropriate controls.

### Further studies of UML-based metabarcoding analysis

This study examined novel UML metabarcoding analysis methods to address specific challenges in DNA metabarcoding analysis. To evaluate their utility, the UML-based methods were compared with OTU clustering and ASV analysis concerning relative abundance, including Procrustes analysis, and detected species, including sensitivity, precision, and F1 score, using datasets derived from the same studies used to develop the methods. The results indicated that one UML-based method (HDBSCAN) was particularly effective for specific applications, and it emerged as the optimal clustering method for NAC or CKSNAP. OTU clustering groups sequences based on similarity thresholds, whereas ASV analysis resolves fine-scale sequence variations, such as single-nucleotide differences [19,25]. Conversely, the UML-based framework proposed in this study incorporates biological feature descriptors and density-based clustering as an integrated analytical approach for analyzing complex DNA metabarcoding datasets. These differences in sequence representation and clustering principles may contribute to the different clustering and taxonomic outcomes observed among the methods. In particular, HDBSCAN identifies clusters based on density structure in the feature space and can accommodate clusters with varying densities and irregular shapes [48,50]. From a methodological perspective, this flexibility suggests that HDBSCAN may be advantageous when sequence-derived feature spaces contain such heterogeneous cluster structures; however, whether these characteristics directly explain its performance in metabarcoding datasets remains to be validated. Similarly, a recent benchmark of environmental metabarcoding datasets showed that the optimal feature-selection approach in machine learning workflows depended on dataset characteristics [44]. The suitability of each method depends on the characteristics of the dataset and the specific research objectives [19,30].

This study focused on the development and validation of UML-based methods, aiming to ensure broad applicability across diverse scenarios by utilizing various markers and targets. The methods were systematically evaluated using mock community data, which provided a controlled environment for assessing their accuracy and performance. However, a major limitation of this study was the lack of application to environmental datasets, which typically present greater complexity and variability than mock community experiments.

In particular, the mock community datasets used in this study feature inherently low ecological complexity, with relatively balanced species composition and limited stochastic variation. Conversely, real environmental samples typically exhibit highly uneven abundance distributions, PCR amplification bias, chimeric artifacts, and intraspecific polymorphism. These factors can substantially affect clustering performance and potentially lead to different outcomes when applying density-based algorithms such as HDBSCAN and OPTICS. Therefore, caution is required when extrapolating the present results to biodiversity monitoring applications based on environmental samples.

To address this limitation, future research should apply the developed methods to diverse environmental datasets, enabling a comprehensive evaluation of their robustness and generalizability. This next step will validate the reliability of UML-based methods in practical contexts and provide insights into their potential limitations and areas for further improvement. Such efforts will ensure that this framework is thoroughly tested and optimized for complex ecological and environmental analyses.

The biological features extracted in this study were tailored to the specific datasets, and NAC or CKSNAP was identified as the optimal feature based on evaluation metrics. UML-based methods differ from OTU and ASV approaches in that they do not require a predefined sequence-similarity threshold or a run-specific error model and can accommodate nonuniform density distributions. However, these capabilities require a higher computational cost. Runtimes measured for the lowest- and highest-dimensional feature descriptors (NAC and CKSNAP, respectively) varied substantially depending on feature dimensionality and dataset size, with OPTICS consistently requiring markedly greater computational resources than HDBSCAN (by a factor of 5 or greater in some cases), further supporting HDBSCAN as the more practical algorithm for large-scale metabarcoding applications (Table S24). Based on these observations, we recommend HDBSCAN combined with a low-dimensional descriptor as a practical default for datasets involving millions of reads, whereas OPTICS and high-dimensional descriptors are better reserved for smaller datasets or analyses in which their additional resolution is specifically required. However, actual runtime and memory requirements may vary depending on dataset characteristics, parameter settings, software implementation, and hardware configuration. Two directions for further study are being considered to address these scalability limitations. First, biologically informed feature selection will be explored to reduce feature dimensionality. Several features currently used contain redundant information arising from the physicochemical properties of DNA, such as base complementarity, and systematic elimination of such redundancy would decrease computational cost without loss of biologically meaningful information. Second, computationally efficient alternatives, including heuristic or approximate clustering algorithms, will be evaluated to reduce time complexity while maintaining clustering quality.

An advantage of UML-based methods is that they do not require a user-defined sequence-similarity threshold or a run-specific error model, which can simplify the analytical process. Biodiversity monitoring using DNA metabarcoding aims to identify the organisms present in a target environment and evaluate their diversity and composition [79]. By applying unsupervised clustering without requiring a predefined sequence-similarity threshold or number of clusters, the UML-based methods provide an additional analytical perspective that complements existing approaches. However, they should not be considered replacements for OTU- and ASV-based approaches, but rather complementary analytical options. Finally, although this study highlighted the potential of UML-based methods, the results underscore the importance of refining experimental and analytical methods. To improve the accuracy of biodiversity analysis using UML-based methods, future research should construct heterogeneous datasets collected from various locations over a sufficiently long period. Such efforts would enhance the applicability of UML-based methods in replicating natural environments and increase their utility for biodiversity monitoring.

## Conclusion

In conclusion, this study developed new UML-based methods for metabarcoding data and compared them with OTU clustering and ASV analysis. According to the results, NAC was the optimal biological feature for UML-based methods, consistently producing well-separated and compact clusters based on geometric structure, as assessed by internal clustering metrics. In comparisons of relative abundance and detected species, HDBSCAN, as a UML-based method, effectively balanced sensitivity and precision under controlled mock community conditions. However, these findings were obtained using mock communities, and the performance of the method on real environmental samples, which exhibit uneven abundance distributions, amplification bias, intraspecific polymorphism, and sequencing artifacts, remains to be validated. Within these limits, UML-based methods may serve as complementary clustering approaches to OTU and ASV analyses under controlled conditions.

## Data Availability

The summary of data analysis is provided in the Supplementary Materials.

## Supplementary Materials

Supplementary 1 Supplementary Materials and Methods Figs. S1 to S12 Tables S1 to S24 References [80–88]
