# Supplementary material for: A New Unsupervised Machine Learning Method for Metabarcoding Analysis Based on Selected Mock Community Data
Source: Comput Struct Biotechnol J. 2026 Sep 22;35(1):0221. doi: 10.34133/csbj.0221 (PMC13594419; doi:10.34133/csbj.0221)
Supplement: Supplementary file 1 [file csbj.0221.f1.docx]

**Supplementary Materials and Methods**

*A New Unsupervised Machine Learning Method for Metabarcoding Analysis Based on Selected Mock Community Data*

# S1. Dataset Construction

## S1.1 Literature Search Strategy

A comprehensive literature search was conducted using Google Scholar in April 2024. The following search terms were used: *"DNA metabarcoding, mock community, OTU"*, *"DNA metabarcoding, mock community, ASV"*, *"12S rRNA, metabarcoding, mock community"*, *"COI, metabarcoding, mock community"*, and *"18S rRNA, metabarcoding, mock community"*. Search results were manually curated by reviewing titles, abstracts, and full texts as necessary. Studies were included only if they (i) employed mock communities with known species identities (i.e., the taxonomic identity of each constituent species was disclosed, although abundance ratios were not necessarily provided by all studies), (ii) targeted 12S rRNA, COI, or 18S rRNA markers, (iii) provided raw sequencing data (FASTQ format) in a publicly accessible repository, and (iv) analyzed data using OTU clustering or ASV analysis. Studies were excluded if they used barcoding for single-species detection, conventional PCR, quantitative PCR (qPCR), or digital PCR (dPCR). From these criteria, two independent studies were identified per marker, yielding six datasets in total (**Table S1**).

## S1.2 Computational Infrastructure

All bioinformatic analyses were executed on an on-premises high-performance computing (HPC) cluster running Rocky Linux 8.7 (Green Obsidian), managed by OpenPBS v22.05.11. The cluster comprised one master node equipped with dual Intel® Xeon® Silver 4110 processors (2.10 GHz, 16 cores total, 62.6 GB RAM) and five dual-socket compute nodes (node01–node04 and gpunode), each equipped with dual Intel® Xeon® Gold 6326 processors (2.90 GHz, 32 cores total, 503 GB RAM per node).

# S2. Sequence Processing and Representative Sequence Generation

## S2-1. OTU- and UML-based Analysis

OTU clustering and UML-based analysis shared an identical preprocessing pipeline. Preprocessing was performed using QIIME2 version 2024.2.0 [54] in conjunction with VSEARCH version 2.22.1 [55] and cutadapt version 4.6 [56]. Read quality was assessed using FastQC version 0.11.9 [80] prior to any processing steps. The following parameters were applied sequentially to both methods.

**Quality trimming:** Bases with a Phred quality score below Q30 were trimmed from the 3′ end of reads. Q30 was applied as it corresponds to a per-base error probability of 0.1% and is widely recognized as a benchmark for base-call accuracy in next-generation sequencing [81].

**Adapter and primer removal:** Adapter and primer sequences were removed using cutadapt version 4.6. Remaining amplicons were filtered using QIIME2 with a maximum expected error rate of 0.2; reads retaining unknown or ambiguous bases (N) were excluded.

**Paired-end read merging:** Forward and reverse reads were merged using VSEARCH version 2.22.1. A minimum base overlap of 12 bp was required for successful merging. The minimum overlap of 12 bp was set to match the default parameter of the DADA2 denoise-paired command (--p-min-overlap 12), ensuring methodological consistency across all three pipelines.

**Low-quality sequence filtering:** Merged sequences were truncated at positions where three or more consecutive bases had a quality score below Q30. Sequences in which truncation exceeded 25% of the total read length, or which contained any ambiguous bases after truncation, were discarded. The 25% truncation threshold was empirically determined to balance sequence quality and read retention, consistent with the minimum per-read length recommendation for Illumina amplicon quality filtering [82].

**Dereplication:** Identical sequences were collapsed into unique sequence representatives using VSEARCH version 2.22.1.

**Chimera filtering:** Chimeric sequences were identified and removed using the uchime-denovo algorithm implemented in VSEARCH.

Detailed read counts before and after preprocessing for each of the six datasets are provided in **Tables S3–S8**.

**OTU clustering.** Following the shared preprocessing steps described above, sequences were clustered into OTUs using a *de novo* OTU picking approach implemented in QIIME2 version 2024.2.0 [54]. Sequence identity thresholds were set at 98.3% for 12S rRNA and 97% for both COI and 18S rRNA, reflecting the differing resolving power of each marker. Singleton OTUs were excluded from downstream analyses.

**UML-based clustering.** Following the shared preprocessing steps, dereplicated sequences were represented as fixed-length feature vectors using 19 biological feature descriptors implemented in the standalone toolkit iLearn [57], (**Table S2**). These descriptors span four major groups: nucleic acid composition, electron–ion interaction pseudopotentials, autocorrelation and cross-covariance, and pseudo nucleic acid composition, comprising 694 features in total.

Density-based clustering was then performed using two algorithms. HDBSCAN version 0.8.33 [60] was applied with *min_cluster_size* = 2 and *min_samples* = 1; all remaining parameters were left at their defaults. OPTICS [51], implemented via scikit-learn version 1.0.2 [61], was applied with *min_samples* = 2 and Euclidean distance as the metric; all remaining parameters were left at their defaults. In both algorithms, sequences assigned a cluster label of −1 (noise) and singleton clusters were excluded from downstream taxonomic assignment. For each retained cluster, the sequence with the highest read count was selected as the representative sequence for taxonomic assignment. In cases where multiple sequences within a cluster shared identical read counts, the sequence appearing first in the input was selected. Clustering quality was evaluated using three internal metrics: the Silhouette Score, Davies–Bouldin Index (DBI), and Calinski–Harabasz Index (CHI). Mathematical definitions of these metrics are provided below.

**Silhouette Score.** The Silhouette Score for a single sample *i* is defined as Equation 1, where *a(i)* is the mean intracluster distance between sample *i* and all other samples in the same cluster, and *b(i)* is the mean distance between sample *i* and all samples in the nearest neighbouring cluster. The overall Silhouette Score is the mean of *S(i)* over all samples, ranging from −1 to 1. Values above 0.7 indicate robust clustering; values between 0.5 and 0.7 indicate reasonable clustering quality [63].

$$Silhouette Score=\frac{b-a}{max\left( a,b \right)} \left( 1 \right)$$

**Davies–Bouldin Index (DBI).** DBI is defined as Equation 2, where *k* is the number of clusters, Δ(*Xᵢ*) is the average intracluster distance for cluster *i*, and δ(*Xᵢ*, *Xⱼ*) is the distance between cluster centroids *i* and *j*. Lower values indicate better-separated, more compact clusters [64].

$$\mathrm{DBI}=\frac{1}{k}\sum_{i=1}^{k} max\left( \frac{\Delta\left( X_{i} \right)+\Delta\left( X_{j} \right)}{\delta\left( X_{i},X_{j} \right)} \right) \left( 2 \right)$$

**Calinski–Harabasz Index (CHI).** CHI is defined as Equation 3, where *Bₖ* is the between-cluster dispersion, *Wₖ* is the within-cluster dispersion, *N* is the total number of data points, and *k* is the number of clusters. Higher values indicate better-defined clusters with greater intercluster separation relative to intracluster variance [65].

$$\mathrm{CHI}=\frac{B_{k}}{W_{k}}\times\frac{N-k}{k-1} \left( 3 \right)$$

All clustering analyses, including the runtime measurements reported in **Table S24**, were conducted on the compute nodes (Intel® Xeon® Gold 6326, 2.90 GHz, 32 cores, 503 GB RAM per node) of the on-premises HPC cluster described above. Each dataset was processed as an independent job submitted to the PBS queue, with one job per node.

## S2-2. ASV Analysis

ASV inference followed a distinct preprocessing pipeline from OTU and UML-based analyses, as DADA2 incorporates its own error-modeling framework that operates on unmerged paired-end reads. Raw reads were trimmed for bases below Q30, after which data were processed using DADA2 version 2022.2.0 [25] implemented within QIIME2 version 2024.2.0 [54], using the *denoise-paired* command. Error models were learned independently for forward and reverse reads. Truncation lengths were determined per dataset based on read quality profiles inspected via FastQC, as detailed below. Reads containing more than two expected errors in either the forward or reverse read were discarded. Paired-end reads were merged with a minimum overlap of 12 bp. Chimeric sequences were removed using the consensus method. Singleton ASVs were excluded from downstream analyses.

Truncation lengths applied per marker and study were as follows: for 12S rRNA, forward and reverse reads were truncated to 130 bp and 120 bp, respectively, in both Study 1 and Study 2. For COI, truncation lengths were 180 bp (forward) and 150 bp (reverse) in Study 1, and 225 bp (forward) and 210 bp (reverse) in Study 2. For 18S rRNA, truncation lengths were 260 bp (forward) and 210 bp (reverse) in Study 1, and 210 bp for both forward and reverse reads in Study 2.

# S3. Taxonomic Assignment and Comparative Analysis

Taxonomic assignment was performed identically across UML-based, OTU, and ASV-processed sequences. For UML-based analysis, reads from each cluster were extracted using dplyr 1.1.1 in R version 4.2.3; singleton clusters were excluded prior to assignment. The consensus-BLAST function with the q2-feature-classifier plugin of QIIME2 [66] was used for taxonomic assignment at 80% similarity for all three methods, using the default parameter of the classify-consensus-blast function (--p-perc-identity 0.8). Reference databases used were: MitoFish [67] for 12S rRNA sequences, BOLD Systems [68] for COI sequences, and SILVA [69] for 18S rRNA sequences.

Processed sequences from all three methods were normalized to the total read count per sample and aggregated into taxonomic profiles at the order, family, genus, and species levels using R version 4.2.3 and the phyloseq package [70]. Sequences not assignable to a valid taxon—including those not classified as class Actinopteri in the case of 12S rRNA—were excluded from downstream analyses. Taxa annotated as "sp." or "uncultured" were also excluded at the relevant taxonomic level. Relative abundance was calculated within samples at each taxonomic level.

**References for Supplementary Methods**

References cited in this Supplementary Methods section are identical to those listed in the main text reference list.
